# Intraoperative PTH Assay during Minimally Invasive Parathyroidectomy May Be Helpful in the Detection of Double Adenomas and May Minimise the Risk of Recurrent Surgery

**DOI:** 10.1155/2010/178671

**Published:** 2010-12-15

**Authors:** E. Ypsilantis, H. Charfare, W. S. Wassif

**Affiliations:** ^1^Princess Royal University Hospital, Farnborough Common, London BR6 8ND, UK; ^2^Bedford Hospital South Wing, Kempston Road, Bedford MK42 9DJ, UK

## Abstract

*Background*. Minimally invasive parathyroidectomy (MIP) is increasingly replacing the traditional bilateral neck exploration in the treatment of primary hyperparathyroidism (PHP). Intraoperative PTH (IOPTH) measurement has recently been introduced as a useful adjunct in confirming successful excision of abnormal parathyroid gland. *Aims*. We evaluate the safety, efficacy, and clinical usefulness of IOPTH measurement during MIP in a district general hospital. *Methods*. Retrospective review of eleven consecutive patients with PHP who underwent MIP with IOPTH, following preoperative assessment with ultrasound and sestamibi scans. *Results*. All patients had successful removal of the abnormal parathyroid gland. The concordance rate between ultrasound and sestamibi scan in localising the parathyroid adenoma was 82%. IOPTH measurement confirmed the removal of adenoma in all cases and, in one case, led to identification of a second adenoma, not localised preoperatively. The median hospital stay was 2 days (range 1–7 days). All patients remained normocalcaemic after a median of 6 months (range 1–10 months). *Conclusions*. Minimally invasive parathyroidectomy is a feasible, safe, and effective method for treatment of PHP. The use of IOPTH monitoring potentially offers increased sensitivity in detecting multiglandular disease, can minimise the need and risk associated with recurrent operations, and may facilitate cost-effective minimally invasive surgery.

## 1. Introduction

Surgery is currently the only available cure for primary hyperparathyroidism (PHP) [1]. In selected patients, minimally invasive parathyroidectomy (MIP) offers the benefits of decreased morbidity and shorter hospital stay; hence, it is increasingly replacing the traditional bilateral neck exploration (BNE) and four-gland evaluation [2]. This technique depends on successful preoperative imaging and localization of the abnormal gland, using ultrasound (USS) and Technetium Tc^99m^ sestamibi scanning with single-photon emission tomography (SPECT) [3]. Using a combination of these imaging modalities, an adenoma could be localised with 95% accuracy. However, the two imaging methods are concordant in only up to 64% of patients with PHP [4], and their accuracy is decreased in multiglandular disease (MGD) [5]. Identification of a macroscopically abnormal parathyroid gland during MIP does not exclude abnormalities of the rest of the glands such as double adenoma, multiglandular disease, or malignant tumours, which may be difficult to distinguish from benign adenomas [6], especially in light of the above limitations of preoperative imaging. On the other hand, histopathology of excised abnormal parathyroid glands is not predictive of the secretory function of the remaining parathyroid glands left in situ [7]. Intraoperative parathyroid hormone (IOPTH) measurement has recently been introduced as a useful adjunct in confirming the successful excision of a parathyroid adenoma. The method is based on the short half-life of the intact PTH (4-5 minutes) [8]; successful removal of diseased parathyroid tissue is predicted by corresponding decrease of serum PTH concentrations. We present our initial experience of patients with PHP treated surgically with minimally invasive parathyroidectomy and IOPTH measurement, and we evaluate the safety, efficacy, and clinical usefulness of the method in a district general hospital.

## 2. Subjects and Methods

The study included eleven consecutive patients (two male and nine female) who underwent surgery for PHP over 6 months period in a district general hospital serving a population of approximately 300,000. The patient characteristics are presented in Table 1. Ten patients were operated as elective cases because of severe hypercalcaemia complicated with decreased bone density. One patient was operated as an emergency due to extreme hypercalcaemia (adjusted serum calcium level, 6.49 mmol/L) with grossly elevated PTH concentration of 1059 pg/mL (reference range 15–65 pg/mL), serum phosphate 1.79 mmol/L, creatinine 124 *μ*mol/L, and urea 16.0 mmol/L [9]. This patient presented acutely with extreme lethargy, dehydration, and drowsiness. Two weeks prior to admission, she had sustained right scaphoid fracture following a fall and was diagnosed with primary hyperparathyroidism (PHPT), awaiting elective parathyroidectomy. All eleven patients had ultrasound scan of the neck preoperatively whereas sestamibi scan was feasible in all but one patient, who was severely kyphotic. Three patients underwent planned full neck exploration facilitated by IOPTH measurements, during the initial period of method validation. In the remaining 8 patients, MIP surgery was initiated under general anaesthesia via a 3 cm skin crease incision approximately 1 cm above the clavicle and centred over the anterior border of sternoceidomastoid muscle. All patients were operated by one surgeon.

Biochemical profile was assayed on a Cobas 6000 analyser (Roche Diagnostics, Mannheim, Germany) using standard methods. Serum calcium was assayed by a method according to Schwarzenbach [10] with o-cresolphthalein complexone and serum albumin was measured by a colourimetric assay [11]. Intact PTH was assayed by a sandwich electrochemiluminescence immunoassay [12]. Intraoperative measurement of serum PTH was performed at baseline (immediately after the adenoma was resected, T0) and then at every 5 minutes post excision of the macroscopically abnormal parathyroid gland for 15 minutes (T5, T10, and T15). A fall in serum PTH level by at least 50% of the baseline value within 15 minutes was used to confirm successful removal of parathyroid adenoma [13].

## 3. Results

**Figure 1:**
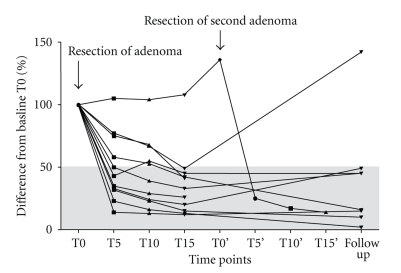
Serum PTH concentrations during the Intraoperative period of minimally invasive parathyroidectomy and at follow up, expressed as percentage difference from the baseline value (T0). T0 represents time of sample taken immediately after resection of adenoma and then at every 5 minutes up to 15 minutes (T5, T10, and T15). T0' represents time of sample taken immediately after resection of a second adenoma and then every 5 minutes for 15 minutes (T5', T10', and T15'). Follow up samples were organised after a median (range) of 6 (1–10) months.

Excision of parathyroid adenoma was followed by the predicted fall in Intraoperative serum PTH concentration of >50% in ten patients. In one patient, the PTH levels increased and remained high for up to 30 minutes post excision, implying the presence of multiglandular disease; further neck exploration identified an ipsilateral second adenoma, not localised preoperatively by either ultrasound or sestamibi scan. Removal of the second adenoma was followed by the expected fall in serum PTH concentration of >50% confirming successful surgery (Figure 1). In another patient, the operation was converted to standard collar incision because the localised parathyroid adenoma macroscopically resembled a thyroid nodule; therefore, better access was deemed necessary; histology confirmed the presence of a parathyroid adenoma only. In all cases, histology confirmed the presence of discrete, encapsulated adenomas, containing sheets and islands of eosenophilic and chief cells, with no capsular invasion. There was no evidence of hyperplasia or carcinoma in either of the eleven patients. During the postoperative period, ten patients had normal PTH and remained normocalcaemic to a median (range) follow up of 6 (1–10) months. Recurrent elevation of serum PTH concentration was noted postoperatively in one patient despite a fall of >50% of baseline Intraoperatively. The patient remains normocalcaemic to date. Two patients had transient clinical hypocalcaemia (with nadir corrected serum calcium levels 2.0 mmol/L and 2.09 mmol/L) and during the first postoperative week requiring calcium and vitamin-D supplementation orally. No other complication was noted. The hospital stay of the elective cases was 2 (1–3) days. The patient who had parathyroidectomy as an emergency when admitted with hypercalcaemic crisis stayed in the hospital for a total of 7 days in order to be monitored for potential complications of extreme hypercalcaemia.

## 4. Discussion

Primary hyperparathyroidism is a common condition. The incidence since the introduction of multichannel analysers is approximately 1 : 1000, with the relatively asymptomatic older female being the typical patient [14]. A prevalence of up to 2.1% in postmenopausal women has been reported in population-based health screening [15]. PHP is predominantly a sporadic disorder [16]. However, in the minority (less than 10%) of affected patients, PHP is associated with a number of distinct hereditary syndromes including multiple endocrine neoplasia (MEN) types 1 and 2A (MEN 1, MEN 2A), hyperparathyroidism-jaw tumour (HPT-JT) syndrome [17], and familial isolated hyperparathyroidism [18]. PHP is primarily caused by benign single adenoma in approximately 80% of patients. Multinodular parathyroid hyperplasia accounts for 15%–20% of cases [19] and only occasionally PHP is caused by parathyroid carcinoma. 

This retrospective study was based on a small preliminary series of patients and is not devoid of the relevant methodological weaknesses, such as incomplete data (e.g preoperative levels of 25-OH vitamin D, urine calcium levels), accounted for by local policies and the retrospective data collection method; these have been addressed in prospective larger series [20–22]. However, it has demonstrated that minimally invasive parathyroidectomy is a safe and effective method of surgical treatment of PHP, constituting a simple procedure that can be carried out in a district general hospital as day surgery or with overnight stay; most of our patients are now discharged within 24 hours with calcium and vitamin-D supplements. Although the role of IOPTH measurement is debatable, in our study, it incurred substantial clinical benefit by identifying the correct pathology in one patient with discordant preoperative imaging and in one patient with double adenoma. It also facilitated successful excision of adenomas without the need for Intraoperative frozen section, which can be disconcordant with the definitive histology in up to 10% of cases [23, 24]. 

Successful outcome of MIP necessitates effective preoperative localization of the abnormal parathyroid tissue. Ultrasound localizes up to 80% of parathyroid adenomas [25]. Tc^99m^ sestamibi scanning, using either single-photon emission computed tomography (SPECT) or oblique pinhole images, is able to localize over 90% of adenomas, including ectopic glands, but is less accurate in multiglandular disease [26]. When ultrasound scan (USS) and Technetium Tc^99m^ sestamibi scanning are concordant their accuracy increases, but this is feasible in only two thirds of patients with PHP (best concordance rate 64%) [27]. In our case series, Intraoperative serum PTH level quantification had higher sensitivity (100%) in detecting the parathyroid pathology (single or double adenoma) compared to each imaging technique alone. Concordance between USS, sestamibi scan and IOPTH was noted in 9 (82%) patients. (Radiologists were not blinded to USS or sestamibi scan). The false negative rate for USS alone was 18% (2 in 11 patients) and for sestamibi scan alone 10% (1 in 10 patients). Combination of all three methods facilitated optimum selection of patients for minimally invasive surgery. 

A major benefit yielded by use of IOPTH measurement in this study was the diagnosis of a double adenoma that had not been detected preoperatively. Double adenomas have been reported to occur in 2%–15% of patients undergoing operation for PHP [28] and can be missed in up to 15% of patients diagnosed with a single adenoma by sestamibi scan [29, 30]. They have a nonuniform distribution, likely to involve both sides of the neck and they can easily be missed when a focused MIP approach is employed. Failure to recognise the double pathology can lead to unsuccessful surgical outcome, unnecessary patient distress, and exposure to the risks of a redo operation and anaesthesia, along with further potential cost. Failure of serum PTH to appropriately decrease after excision of a single adenoma should raise suspicion of additional adenomas and the surgical approach should be modified accordingly, if necessary by converting MIP to a standard full neck exploration. 

Although Intraoperative serum PTH level quantification was first introduced in 1990, it has yet to be universally accepted as a routine adjunct in surgery for PHP. Opponents of the method claim the additional incurring cost of surgery [31], prolongation of the operating time of MIP and the reported false negative rate [32]. We did not perform a cost analysis during this small study, but it is anticipated that the short median hospital stay of our patients was cost effective; the additional cost of PTH assays of *£*32 per patient (4 Intraoperative measurements) has been offset by reducing the hospital stay (cost of *£*657 per overnight stay per patient [33]) and by eliminating the cost of Intraoperative frozen section (*£*150 per patient). The use of IOPTH measurement in our series added 5–10 minutes extra to the operating time of minimally invasive procedure. MIP in experienced hands has been shown to be associated with shorter operating times [34] comparing to the conventional neck exploration, but studies providing high levels of evidence are lacking in this respect. 

One patient in our study was found to have elevated serum PTH during follow up, despite appropriate Intraoperative PTH fall after excision of adenoma, but remains normocalcaemic and asymptomatic; hence, no further imaging has been deemed clinically necessary. This phenomenon is not uncommon and its significance is uncertain; between 8%–40% of patients with normocalcemia after surgery have elevated PTH levels at follow up [35, 36]. In a recent study, Ning et al. [37] demonstrated that 95% of these patients do not develop recurrent hyperparathyroidism (RHP). Proposed potential risk factors include higher preoperative PTH concentrations, higher alkaline phosphatase activities, and older age (associated with impaired renal function, bone remineralization, and low vitamin D). They supported the hypothesis that persistently elevated PTH may be, in part, secondary to “bone hunger” and suggested that RHP in their series was more likely if postoperative serum calcium was higher than 9.7 mg/dL (2.42 mmol/L). Long follow up with yearly serum PTH and calcium levels is necessary to detect potential recurrence at an early stage.

In summary, the results of our small case series suggest that minimally invasive parathyroidectomy with Intraoperative PTH measurement is a feasible, safe, and effective method for treatment of primary hyperparathyroidism in the context of a district general hospital. Our findings are in accordance with similar studies appealing for its wider use in the UK [38].

##  Declaration of Interest

There is no conflict of interest that could be perceived as prejudicing the impartiality of the research reported.

##  Funding

This research did not receive any specific grant from any funding agency in the public, commercial or not-for-profit sector.

## Figures and Tables

**Table 1:** Patient characteristics, preoperative and postoperative serum calcium, and parathormone levels.

Patient characteristics				
	Median	Range		
Age				

Serum vitamin D (nmol/L)	61	46–67		
	26	16–35		
Gender	N	%		

* Male*	2	18		
* Female*	9	82		
Operation				

*Elective *	10	91		
*Emergency *	1	9		
Patients	Preop PTH (pg/mL)	Preop serum Ca (mmol/L)	Postop PTH (pg/mL)	Postop serum Ca (mmol/L)

*Pt 1*	154	3.13	75	2.63
*Pt 2*	1059	6.06	28	2.32
*Pt 3*	1924	3.61	162	2.23
*Pt 4*	86	2.70	43	2.32
*Pt 5*	44	3.07	20	2.33
*Pt 6*	697	3.82	14	2.25
*Pt 7*	142	2.91	68	2.26
*Pt 8*	367	4.00	10	2.85
*Pt 9*	194	2.90	92	2.29
*Pt 10*	249	3.14	42	2.3
*Pt 11*	120	2.90	47	2.45

## Abbreviations

IOPTH: Intraoperative parathyroid hormone
MIP: Minimal invasive parathyroidectomy
PHP: Primary hyperparathyroidism
BNE: Bilateral neck exploration
USS: Ultrasound scan
SPECT: Single photon emission tomography
MGD: Multiglandular disease
MEN: Multiple endocrine neoplasia
HPT-JT: Hyperparathyroidism-jaw tumour.

## References

[1] Palazzo FF, Delbridge LW (2004). Minimal-access/minimally invasive parathyroidectomy for primary hyperparathyroidism. *Surgical Clinics of North America*.

[2] Howe JR (2000). Minimally invasive parathyroid surgery. *Surgical Clinics of North America*.

[3] Chen H, Mack E, Starling JR (2005). A comprehensive evaluation of perioperative adjuncts during minimally invasive parathyroidectomy: which is most reliable?. *Annals of Surgery*.

[4] Coakley AJ, Kettle AG, Wells CP, O’Doherty MJ, Collins REC (1989). 99Tcm sestamibi—a new agent for parathyroid imaging. *Nuclear Medicine Communications*.

[5] Perrier ND, Ituarte PHG, Morita E (2002). Parathyroid surgery: separating promise from reality. *Journal of Clinical Endocrinology and Metabolism*.

[6] Conroy S, Wassif WS (2003). Hyperparathyroidism—pitfalls in management. *British Journal of Biomedical Science*.

[7] Carneiro-Pla DM, Romaguera R, Nadji M, Lew JI, Solorzano CC, Irvin GL (2007). Does histopathology predict parathyroid hypersecretion and influence correctly the extent of parathyroidectomy in patients with sporadic primary hyperparathyroidism?. *Surgery*.

[8] Bieglmayer C, Prager G, Niederle B (2002). Kinetic analyses of parathyroid hormone clearance as measured by three rapid immunoassays during parathyroidectomy. *Clinical Chemistry*.

[9] Kankara CR, Fleming E, Morrish N, Melvin A, Charfare H, Wassif WS (2008). Surviving extreme hypercalcaemia. *Clinical Endocrinolgy*.

[10] Schwarzenbach G (1955). The complexones and their analytical application. *The Analyst*.

[11] Doumas BT, Watson WA, Biggs HG (1971). Albumin standards and the measurement of serum albumin with bromcresol green. *Clinica Chimica Acta*.

[12] Blind E, Schmidt-Gayk H, Armbruster FP, Bouillon R (1990). Measurment of intact parathyroid hormone by an extracting two-site immunometric assay. *Calcuim Regulating Hormones, Vitamin D Metabolites, an Cyclic AMP*.

[13] Robertson GSM, Iqbal SJ, Bolia A, Bell PRF, Veitch PS (1992). Intraoperative parathyroid hormone estimation: a valuable adjunct to parathyroid surgery. *Annals of the Royal College of Surgeons of England*.

[14] Conroy S, Moulias S, Wassif WS (2003). Primary hyperparathyroidism in the older person. *Age and Ageing*.

[15] Lundgren E, Rastad J, Thurfjell E, Åkerström G, Ljunghall S (1997). Population-based screening for primary hyperparathyroidism with serum calcium and parathyroid hormone values in menopausal women. *Surgery*.

[16] Mallette L, Bilezikian JP, Levine M, Marcus R (1994). The functional and pathologic spectrum of parathyroid abnormalities in hyperparathyroidism. *The Parathyroids: Basic and Clinical Concepts*.

[17] Wassif WS, Farnebo F, Teh BT (1999). Genetic studies of a family with hereditary hyperparathyroidism-jaw tumour syndrome. *Clinical Endocrinology*.

[18] Wassif WS, Moniz CF, Friedman E (1993). Familial isolated hyperparathyroidism: a distinct genetic entity with an increased risk of parathyroid cancer. *Journal of Clinical Endocrinology and Metabolism*.

[19] Wassif WS, East J, Marshall WJ, Bangert SK (2008). Metabolic effects of tumours. *Clinical Biochemistry Metabolic and Clinical Aspects*.

[20] Chan RK, Ibrahim SI, Pil P, Tanasijevic M, Moore FD (2005). Validation of a method to replace frozen section during parathyroid exploration by using the rapid parathyroid hormone assay on parathyroid aspirates. *Archives of Surgery*.

[21] Lo C-Y, Chan W-F, Leung P, Luk JM (2005). Applicability of tissue aspirate for quick parathyroid hormone assay to confirm parathyroid tissue identity during parathyroidectomy for primary hyperparathyroidism. *Archives of Surgery*.

[22] Horanyi J, Duffek L, Szlávik R, Takács I, Tóth M, Romics L (2010). Intraoperative determination of pth concentrations in fine needle tissue aspirates to identify parathyroid tissue during parathyroidectomy. *World Journal of Surgery*.

[23] Irvin GL, Dembrow VD, Prudhomme DL (1991). Operative monitoring of parathyroid gland hyperfunction. *American Journal of Surgery*.

[24] Prey MU, Vitale T, Martin SA (1989). Guidelines for practical utilization of intraoperative frozen sections. *Archives of Surgery*.

[25] Nicholson DA, Dawson P, Lavender JP, Lynn J, Bloom SR (1993). Imaging of the parathyroids. *Surgical Endocrinology*.

[26] Ho Shon IA, Bernard EJ, Roach PJ, Delbridge LW (2001). The value of oblique pinhole images in pre-operative localisation with 99mTc-MIBI for primary hyperparathyroidism. *European Journal of Nuclear Medicine*.

[27] Borley NR, Collins REC, O’Doherty M, Coakley A (1996). Technetium-99m sestamibi parathyroid localization is accurate enough for scan-directed unilateral neck exploration. *British Journal of Surgery*.

[28] Abboud B, Sleilaty G, Helou E (2005). Existence and anatomic distribution of double parathyroid adenoma. *Laryngoscope*.

[29] Genc H, Morita E, Perrier ND (2003). Differing histologic findings after bilateral and focused parathyroidectomy. *Journal of the American College of Surgeons*.

[30] Lee NC, Norton JA, Dainko EA, Duh QY (2002). Multiple-gland disease in primary hyperparathyroidism: a function of operative approach?. *Archives of Surgery*.

[31] Agarwal G, Barakate MS, Robinson B (2001). Intraoperative quick parathyroid hormone versus same-day parathyroid hormone testing for minimally invasive parathyroidectomy: a cost-effectiveness study. *Surgery*.

[32] Miura D, Wada N, Arici C, Morita E, Duh QY, Clark OH (2002). Does intraoperative quick parathyroid hormone assay improve the results of parathyroidectomy?. *World Journal of Surgery*.

[33] Coughlin SM, Walker I, Wassif WS (2008). The impact of provision of extended laboratory service for Troponin T assay on hospital admission. *Annals of Clinical Biochemistry*.

[34] Bergenfelz A, Lindblom P, Tibblin S, Westerdahl J (2002). Unilateral versus bilateral neck exploration for primary hyperparathyroidism: a prospective randomized controlled trial. *Annals of Surgery*.

[35] Denizot A, Pucini M, Chagnaud C, Botti G, Henry JF (2001). Normocalcemia with elevated parathyroid hormone levels after surgical treatment of primary hyperparathyroidism. *American Journal of Surgery*.

[36] Wang TS, Ostrower ST, Heller KS (2005). Persistently elevated parathyroid hormone levels after parathyroid surgery. *Surgery*.

[37] Ning LI, Sippel R, Schaefer S, Chen H (2009). What is the clinical significance of an elevated parathyroid hormone level after curative surgery for primary hyperparathyroidism?. *Annals of Surgery*.

[38] Palazzo FF, Sadler GP, Reene TS (2004). Minimally invasive parathyroidectomy: heralds a new era in the treatment of primary hyperparathyroidism. *British Medical Journal*.

